# Editorial: Children and young people's mental health in a “post-pandemic” age

**DOI:** 10.3389/fpsyg.2024.1447398

**Published:** 2024-06-26

**Authors:** Lisa Chiara Fellin, Valentina Fantasia, Jane E. M. Callaghan

**Affiliations:** ^1^Department of Human and Social Sciences, University of Bergamo, Bergamo, Italy; ^2^Department of Philosophy and Cognitive Science, Lund University, Lund, Sweden; ^3^Centre for Child Wellbeing and Protection, University of Stirling, Stirling, United Kingdom

**Keywords:** CAMH, children and young people (CYP), mental health, COVID-19, post-pandemic era, systemic impacts, wellbeing, children's rights

Whilst the short-term and most evident impact of the COVID-19 pandemic in the early 2020s involved physical illness and its direct consequences, its long-term, systemic impact was, and still is, deep and wide-ranging. In particular the long-term impact of the COVID-19 pandemic on children and young people's mental health and wellbeing has been dramatic (Levante et al.).

Since the beginning of this dramatic pandemic, a growing amount of research has been devoted to investigate how prolonged periods of “lockdown” have affected the educational, emotional, and social wellbeing of children and young people, in both the short and long term.

In this Research Topic, eight research articles adopting a variety of different methods and target groups, spanning from China to the USA, explored how children and young people across the world coped with the pandemic, its impact on their physical and mental health and wellbeing, as well as protective factors involving their closest social networks, from parents' and family functioning to peer interactions.

Levante et al. present a systematic review of 34 papers published during 2020–2022 involving 40,976 participants in total. Their analysis shows that both children's internalizing and externalizing symptoms increased during the pandemic, mostly because of limited play activities and excessive internet use. Parents' distress also appeared as the strongest parental factor mediating children's symptoms. The studies reviewed were cross-sectional, so long-term patterns and outcomes could not be predicted, but this important review sheds light on the impact of the pandemic onto children's wellbeing.

Vieira et al. propose a cross-sectional, multicenter study examining pandemic social isolation-related factors which appeared to contribute to internalizing and externalizing behavior problems in 113 children (already at risk for neurodevelopmental disorders) in Brazil. As the children were high-risk newborns followed-up in tertiary units of the public health system, prematurity appeared to be associated with externalizing problems, and change in eating habits with internalizing problems. The study highlights key protective and risk factors for the emergence of children's behavioral problems, including parents' educational level (at least having completed high school) and child-care duties (both sharing care of the child) as protective factors. Reports of sleeping problems and living with another child emerged as risk factors. The findings confirm the essential role played by family-systems functioning for children's wellbeing and family-centered interventions.

Few studies have investigated the physiological impact of stressful pandemic-related changes to everyday life, especially in children. Lloyd et al. conducted a cross-sectional analysis on 94 preadolescents, showing how restrictions due to pandemic-related lockdown in the Boston area (USA) was linked to dysregulated cortisol (hypocortisolism) and salivary alpha amylase levels. The study highlights how these physiological changes in response to chronic stress in children can have negative consequences on other physiological systems, potentially causing damages affecting mental and physical health and wellbeing.

Four research papers from China analyze the pandemic impact on children in the first area hit by COVID-19.

Zhang et al. investigated the influence of parent–child attachment on school adjustment among the left-behind children of overseas Chinese migrants. This relation seems to be mediated by aspects such as parents' identity and childrens' peer relationships. With an increased number of children left behind who cannot see their parents due to the impact of COVID-19, efforts to facilitate school adjustment and family and social interactions seem extremely important in tackling the additional and unique challenges posed for this population.

Wang et al.'s cross-sectional study investigated the psychological wellbeing and emotion regulation skills of 436 high school students during the COVID-19 pandemic in China.

The quality of adolescents' affective experience appeared to be influenced by multiple emotion-regulation strategies, including cognitive reappraisal, acceptance and engagement, awareness, and modification of emotions, as well as relational and socio-demographic variables.

Li et al. explored depression, anxiety, stress symptoms and their determinants among secondary students with vision impairment in rural Northwestern China during the COVID-19 pandemic, through a cross-sectional study with 1,992 secondary school students. The study revealed that approximately a quarter of children experienced symptoms of depression and stress, and close to half of the sample experienced symptoms of anxiety. The Authors advocate for Healthcare planners in China to consider policies and interventions aiming at reducing recreational screen time, ensuring sufficient sleep, and timely detection of mental health symptoms, particularly among socioeconomically disadvantaged groups who were the most affected.

The outcomes of children infected and uninfected with COVID-19 in Hong Kong were compared by Lau et al.. Their study explored the association between parents' use of mobile phones to calm children in a variety of situations through an online survey completed by 1,187 parents when schools were suspended during the 5th wave of COVID-19 resurgence. They found no substantial differences in various psychological, social, emotional, and behavioral outcomes between infected and uninfected children, suggesting that support during pandemics should be provided to children and families regardless of whether children have been infected with COVID-19.

Russian young people's subjective health evaluations, self-care practices, and therapeutic networks were explored by Mikhaylova through a thematic analysis of semi-structured interviews with 41 Russian youths. She identified that young people who had low health evaluations were more likely to engage in self-care and sports to enhance their health and to have mothers and other medical experts in their therapeutic networks.

In different ways, all articles included in this Research Topic highlight how the chronic stress and the disruptions caused by COVID-19 to daily life, including the stressful experience of community lockdown and isolation, had multiple long-lasting effects on children and young people's physical and mental health, wellbeing, relational life and agency.

These contributions shed light on the dramatic social and family transformations and challenges produced by the pandemic, and their implications for service delivery, family life, and children and young people's outcomes, in particular for children with special needs or disabilities. Given the multiple systemic impacts of the COVID-19 pandemic on children, which found services completely unprepared and unfit, paying close attention to their wellbeing in the longer term is warranted, especially in terms of funding and provision. This includes the intensification of existing health/mental health inequalities at local and global levels, calling for new services and policies in terms of prevention, monitoring and intervention in the short and long term. Future researchers will thus require a longitudinal, multidisciplinary approach to determine the long-term effects of the pandemic on children's psychological and physical wellbeing, e.g., by looking at internalizing and externalizing symptoms, physiological measurements and broader social/relational networking resources. The need for re-thinking and transforming the mental health service landscape to better recognize and respond to the increasing needs of people who use those services has been recently advanced by the Pūras (2020) and WHO (2021). Changes produced by the pandemic crisis, including, for instance, peer based interventions, digital innovations and clinician-led work have introduced new important and inclusive tools to address unmet needs and unforeseen consequences of the pandemic and related lockdown.

The post-pandemic era we live in now may be a unique opportunity to do so, re-designing mental health services, tools and provisions for children and young people in a way that can centralize and empower them.

## Author contributions

LF: Conceptualization, Writing – original draft, Writing – review & editing. VF: Conceptualization, Writing – original draft, Writing – review & editing. JC: Writing – review & editing.
